# S100A12 concentrations and myeloperoxidase activities are increased in the intestinal mucosa of dogs with chronic enteropathies

**DOI:** 10.1186/s12917-018-1441-0

**Published:** 2018-04-04

**Authors:** Mohsen Hanifeh, Satu Sankari, Minna M. Rajamäki, Pernilla Syrjä, Susanne Kilpinen, Jan S. Suchodolski, Romy M. Heilmann, Phillip Guadiano, Jonathan Lidbury, Jörg M. Steiner, Thomas Spillmann

**Affiliations:** 1 Department of Equine and Small Animal Medicine, Faculty of Veterinary Medicine, University of Helsinki, PO Box 57, Viikintie 49, 00014 Helsinki, Finland; 2 Department of Clinical Sciences, Faculty of Veterinary Medicine, University of Tabriz, Tabriz, 5166616471 Iran; 3 Department of Veterinary Biosciences, Faculty of Veterinary Medicine, University of Helsinki, PO Box 66, Agnes Sjöberginkatu 2, 00014 Helsinki, Finland; 4 Gastrointestinal Laboratory, Department of Small Animal Clinical Sciences, College of Veterinary Medicine and Biomedical Sciences, Texas A&M University, College Station, TX 77843–4474 USA; 5 Department of Small Animal Medicine, Veterinary Teaching Hospital, University of Leipzig, An den Tierkliniken 23, 04103 Leipzig, SN Germany

**Keywords:** S100A12, Myeloperoxidase, Chronic enteropathies, Dog

## Abstract

**Background:**

Intestinal mucosal S100A12 and myeloperoxidase (MPO) are inflammatory biomarkers in humans with inflammatory bowel disease (IBD). However, these biomarkers have not been studied in the intestinal mucosa of dogs with chronic enteropathies (CE), even though dogs with CE have increased S100A12 concentrations in feces and serum. This study investigated mucosal S100A12 concentrations and MPO activities in both dogs with CE and healthy Beagles. ELISA (S100A12 concentrations) and spectrophotometric methods (MPO activity) were used. The associations of both biomarkers with canine IBD activity index (CIBDAI), histopathologic findings, clinical outcome, and serum albumin concentrations were also investigated. We studied intestinal mucosal samples originating from different intestinal regions of 40 dogs with CE and 18 healthy Beagle dogs (duodenum, ileum, colon, and cecum).

**Results:**

Compared with healthy Beagles, mucosal S100A12 concentrations in dogs with CE were significantly higher in the duodenum (*p* < 0.0001) and colon (*p* = 0.0011), but not in the ileum (*p* = 0.2725) and cecum (*p* = 0.2194). Mucosal MPO activity of dogs with CE was significantly higher in the duodenum (*p* < 0.0001), ileum (*p* = 0.0083), colon (*p* < 0.0001), and cecum (*p* = 0.0474). Mucosal S100A12 concentrations in the duodenum were significantly higher if the inflammatory infiltrate consisted mainly of neutrophils (*p* = 0.0439) or macrophages (*p* = 0.037). Mucosal S100A12 concentrations also showed a significant association with the severity of total histopathological injury and epithelial injury in the colon (*p* < 0.05). Mucosal MPO activity showed a significant association (*p* < 0.05) with the severity of total histopathological injury, epithelial injury, and eosinophil infiltration in the duodenum. There was no significant association of both biomarkers with CIBDAI or clinical outcome.

**Conclusions:**

This study showed that both mucosal S100A12 concentrations and MPO activities are significantly increased in the duodenum and colon of dogs with CE; mucosal MPO was also increased in the ileum and cecum. Future research should focus on assessing the clinical utility of S100A12 and MPO as diagnostic markers in dogs with CE.

## Background

Canine chronic enteropathy (CE) is a group of inflammatory conditions of the intestinal tract with unknown etiology [1]. Based on treatment response, canine CE is defined as food-responsive diarrhea or enteropathy (FRD or FRE), antibiotic-responsive diarrhea or enteropathy (ARD or ARE), steroid-responsive diarrhea or enteropathy (SRD or SRE), or steroid-non-responsive diarrhea or enteropathy (SNRD or SNRE) [2, 3]. While the etiology and pathogenesis of canine CE is not fully understood, an aberrant immune response to antigens derived from endogenous microbiota and diet is likely to play an important role in canine CE pathogenesis [2, 4, 5]. Therefore, phagocyte activation biomarkers may represent potential and useful markers of inflammation in dogs with CE. Accordingly, more research is needed to clarify the roles of these markers in the pathogenesis of CE.

S100A12 (also known as calgranulin C) belongs to the S100/calgranulin protein subfamily and is mainly expressed and secreted by activated neutrophils [6, 7] and macrophages/monocytes [8]. *After S100A12 release* into the extracellular space, either due to cell damage or activation of phagocytes, *S100A12* acts as a ligand for the advanced glycation end products (RAGE) receptor [9, 10]. Binding to RAGE can induce sustained post-receptor signaling, including activation of nuclear factor κB (NF-κB) and the upregulation of transmembrane RAGE itself which can in turn lead to amplification and perpetuation of the inflammatory response [9–11]. S100A12 is a useful biomarker in human patients with inflammatory diseases, such as IBD [12–23]. S100A12 concentrations are increased in fecal samples, serum, and intestinal mucosa from human patients with IBD [12–19]. S100A12 concentrations are also increased in feces and serum from dogs with CE [24–26]. In addition, increased fecal S100A12 concentrations in dogs with CE correlate with severity of clinical signs, endoscopic lesions in the duodenum, colonic inflammation, and negative outcome [24, 25]. Fecal S100A12 concentrations have also been measured in dogs with different types of CE, including FRD, ARD, SRD, and SNRD [26]. Increased fecal S100A12 concentrations have been reported in dogs with SRD when compared with dogs with FRD or ARD, but also in SNRD dogs when compared to dogs in complete remission after steroid treatment [26]. However, when measuring fecal S100A12 concentrations, it is not possible to differentiate the region of origin within the intestinal mucosa. Given the various physiological and pathophysiological roles of S100A12, it is reasonable to consider this protein’s function in the intestinal mucosa during inflammation in dogs with CE.

Myeloperoxidase (MPO) is an enzyme found in neutrophils and at lower concentrations in macrophages/monocytes and eosinophils [27–29]. Myeloperoxidase generates hypochlorous acid (HOCl) from hydrogen peroxide (H_2_O_2_) and chloride and plays an important role in intracellular microbial destruction [27–29]. However, after phagocyte activation at an inflammatory site, MPO is released into the extracellular space and induces oxidative tissue damage of host tissue [30]. Increased mucosal MPO activity can be used as a biomarker of oxidative stress and has been described in human patients with IBD (both Crohn’s disease [CD] and ulcerative colitis [UC]) [31–34] and also in animal models of human IBD [35–37]. In our previous study, we measured mucosal S100A12 concentrations and MPO activities in different segments of the intestine of healthy Beagles (duodenum, jejunum, ileum, and colon) [38]. However, to our knowledge, intestinal mucosal S100A12 concentrations and MPO activities have not yet been investigated in dogs with CE.

We hypothesized that mucosal S100A12 concentrations and MPO activities are increased in dogs with CE compared with healthy Beagles. We also assessed for any possible association of intestinal mucosal S100A12 concentrations and MPO activities with histopathological findings, canine inflammatory bowel disease activity index (CIBDAI) scores, clinical outcome, or hypoalbuminemia in dogs with CE.

## Methods

### Study population

#### Dogs with chronic gastrointestinal signs

**Fig. 1:**
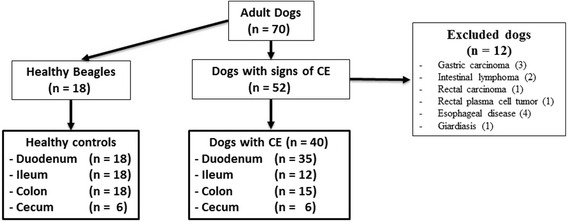
Flow diagram of enrolled dogs. Flow diagram showing group distribution and inclusion and exclusion criteria of all dogs enrolled in the study. CE: chronic enteropathies

A total of 52 dogs with chronic gastrointestinal (GI) signs such as vomiting, diarrhea, tenesmus, hematochezia, or weight loss lasting more than 3 weeks were enrolled into our study over a 4-year period. For each dog, diagnostic tests were performed to exclude underlying gastrointestinal infections (e.g. giardiasis) and neoplasia or extraintestinal disorders. These tests included complete blood count, serum biochemical analysis, fecal examination for parasites, abdominal ultrasound, and gastroduodenoscopy or colonoscopy (or both) with biopsy and histopathological examination. All the tests were performed at the Veterinary Teaching Hospital, Faculty of Veterinary Medicine, University of Helsinki, Finland. The diagnosis of chronic enteropathy (CE) was based on previously published clinical, laboratory, endoscopic, and histopathologic criteria [39, 40]. Before starting any treatment, all dogs with chronic GI signs underwent endoscopic examination. The area of endoscopy was selected based on the clinical signs. Intestinal mucosal biopsies from dogs with chronic GI signs were collected over a 4-year period and were stored at − 80 °C for 1-4 years for S100A12 and MPO determinations. The flow diagram (Fig. 1) shows the group distribution of enrolled dogs into the study.

#### Healthy beagles

Intestinal tissue samples from 18 healthy laboratory Beagle dogs were used as controls. The samples were collected during post-mortem examinations at the conclusion of unrelated studies. The Beagle dogs were housed according to the European Union guidelines in indoor groups with access to outdoor runs. The indoor environmental temperature was maintained between 15 °C and 24 °C. The dogs were exposed to both natural and artificial light (from 7 AM to 4 PM) and were fed a standard commercial diet. All dogs were considered healthy based on history, physical examination, complete blood count, serum biochemistry profile, fecal examination, and histologic evaluation. Intestinal mucosal samples were collected from four different segments of the intestine. From all dogs (*n* = 18) duodenum, ileum, and colon samples were provided; cecum samples were obtained from six dogs (Fig. 1). All intestinal samples from healthy Beagles were taken immediately after euthanasia, snap-frozen in liquid nitrogen and stored at − 80 °C for 4-7 years and 1-4 years for S100A12 and MPO determinations, respectively.

### Ethical statement

The clinical trial involving dogs with CE was prospectively planned and all procedures were carried out under an ethical approval granted by the Finnish National Animal Experiment Board (study license No. ESAVI/6973/04.10.03/2011 and ESAVI/10384/04.10.07/2014). Informed owner consent was obtained at the time the dogs were enrolled for gastroduodenoscopy, colonoscopy, or both. The samples from healthy Beagles were collected during post-mortem examinations after finalizing unrelated studies. These studies were approved by the Finnish National Animal Experiment Board (study license No. ESLH-2007-09833/Ym-23, ESAVI 2010-04178/Ym-23, and ESAVI/7290/04.10.03/2012). All sections of this report adhere to the ARRIVE Guidelines for reporting animal research [41]. A completed ARRIVE guidelines checklist is included in Checklist S1.

### Clinical disease activity in dogs with chronic enteropathies

The clinical disease activity in dogs with CE was determined based on the CIBDAI scoring system at the time of study start and after treatment [42]. CIBDAI was assessed using six prominent GI signs (ie, attitude and activity, appetite, vomiting, stool consistency, stool frequency, and weight loss); scores are based on severity and range from 0 to 3. The total CIBDAI score represents the sum of all individual scores and was classified as insignificant (score 0-3), mild (score 4-5), moderate (score 6-8), or severe (score ≥ 9). Recording the CIBDAI score before and after treatment was only possible in 30 of 40 dogs with CE, and was based on either available scores taken by the responsible clinician before and after treatment (in 13/30 and 5/30 of dogs, respectively) or calculated retrospectively by the investigators (in 17/30 and 25/30 of dogs, respectively). For retrospectively calculated scores, information was obtained from clinical history (before treatment) and phone interviews with the owners (after treatment). The treatment follow up of patients were not based on a standardized time frame and the second CIBDAI was either based on control visits or phone calls at least 2 weeks apart from the start of the treatment. The CE type was determined by response to treatment and since not all included dogs developed diarrhea as a clinical sign, the CE type was defined as FRE, ARE, SRE, and SNRE [2, 3]. As antibiotic, Tylosin at a dose of 25 mg/kg/day for 7 days was mainly used [43]. In some canine patients also metronidazole with/without enrofloxacin was used. For all three SNRE dogs, anallergenic diet (Royal Canin®) was started first, followed by antibiotic trial and consecutive prednisolone in two, but immediate prednisolone in the third dog. All owners opted for euthanasia due to severity of clinical signs and unfavorable response.

### Serum albumin concentrations in dogs with chronic enteropathies

The serum albumin concentration was determined in each dog with CE. A serum albumin concentration < 20 g/L was considered hypoalbuminemic [1].

### Histopathological examination

Histopathological assessment of mucosal tissue samples from canine patients and healthy Beagles collected by endoscopy or during necropsy was performed. The samples were fixed in 4% formaldehyde solution in phosphate-buffered saline, embedded in paraffin, sectioned (3-5 μm), and stained with hematoxylin and eosin (HE). Histopathological changes of the samples were evaluated and scored by a single pathologist (PS) using the guidelines of the World Small Animal Veterinary Association (WSAVA) Gastrointestinal Standardization Group [39, 40]. In every case, the pathologist was blinded to the results of clinical and laboratory examinations and to mucosal levels of S100A12 and MPO. The severity of histopathological changes in different regions of the intestine was evaluated and scored as normal (0), mild (1), moderate (2), or severe (3) according to the WSAVA standardization guidelines. The total histopathological injury score was defined as the sum of the morphology and inflammatory scores and was classified as insignificant (total score 0-4), mild (total score 5-9), moderate (total score 10-14), severe (total score 15-19), or very severe (total score ≥ 20). The samples from ileum and cecum were examined and scored using the guidelines provided for the interpretation of duodenal and colonic biopsies, respectively; this was performed because of the absence of specific templates for these intestinal segments in the WSAVA Gastrointestinal Standardization Group guidelines [39, 40].

### Measurement of mucosal S100A12 concentration

Snap-frozen intestinal mucosal samples from dogs with CE and healthy Beagles were homogenized using a Polytron homogenizer in ice-cold extraction buffer (containing 50 mM Tris/HCl base, 150 mM NaCl, 10 mM CaCl_2_, 0.2 mM NaN_3_ and 0.01% (*v*/v) Triton X-100; pH 7.6) in the presence of EDTA-free protease inhibitor cocktail tablets (1 tablet/10 ml of extraction buffer) at a 20:1 ratio of extraction buffer to tissue. After homogenization, samples were centrifuged at 13000 *g* at 4 °C for 10 min. Supernatants were collected and stored at − 80 °C until assayed for S100A12 concentration. S100A12 concentrations were measured using an ELISA method as previously described [38, 44]. In brief, immunoassay plates were coated with 200 ng/well of affinity-purified polyclonal anti-canine S100A12 antibody and were blocked with 25 mM Tris-buffered saline (TBS), 150 mM NaCl, 0.05% (v/v) polyoxyethylene-20 sorbitan monolaurate, and 10% (*w*/*v*) bovine serum albumin (BSA) at pH 8.0. Plates were then incubated with duplicates of standard purified canine S100A12 solutions, assay controls, or mucosal extracts diluted in 25 mM TBS, 150 mM NaCl, 0.05% polyoxyethylene-20 sorbitan monolaurate, and 0.5% (w/v) BSA at pH 8.0. To detect captured antigen, plates were incubated with horseradish peroxidase-labeled anti-canine S100A12 polyclonal antibody (15 ng/well) and developed with a stabilized 3,3′,5,5′-tetramethylbenzidine substrate (TMB). After a 5-min incubation with TMB, color development was stopped with 4 M acetic acid and 0.5 M sulfuric acid. Absorbance was measured at 450 nm. The researcher (PG) was blinded to the individual dogs’ clinical data (ie, health status, clinical outcome, and intestinal segment). The lower detection limit of the ELISA assay for canine S100A12 was determined by calculating the mean response + 3 times the standard deviation (SD) for 20 replicates of the blank solution. S100A12 concentrations in extracts of snap-frozen intestinal mucosal tissues were measured using the same lot of reagents for all samples and are reported as μg/L of the intestinal mucosal supernatant.

### Measurement of mucosal myeloperoxidase activity

To measure mucosal MPO activity, snap-frozen intestinal mucosal samples from healthy Beagles and dogs with CE were weighed, suspended in ice-cold extraction buffer at a 20:1 ratio of extraction buffer to tissue, and homogenized as previously described [38]. After homogenization, samples were centrifuged at 4000 *g* at 4 °C for 20 min. The supernatants were collected and stored at − 80 °C. MPO activity was determined in intestinal mucosa as previously described [38]. Briefly, 5 μL of supernatant and 5 μL of distilled water were added to the reaction mixture containing 170 μL of sodium phosphate buffer (80 mM, pH 5.4) with hexadecyltrimethylammonium bromide (HTAB, 0.5% *w*/*v*) and 3,3′,5,5′-tetramethylbenzidine (1.6 mmol/L). The mixture was then incubated for 6 min at 37 °C, after which 20 μL of H_2_O_2_ (0.3 mmol/L) was added. After the addition of H_2_O_2_, a kinetic measurement for 60 s was started at a wavelength of 620 nm using an automated biochemical analyzer (Konelab 30i, Thermo Fisher Scientific, Vantaa, Finland). The researchers performing the laboratory analysis (MH and SS) were blinded to the individual dog’s clinical data (ie, health status, clinical outcome, and intestinal segment). The lower detection limit of the spectrophotometric assay for mucosal MPO was calculated based on the following formula: mean blank + 3 × SD for 7 replicates of the blank solution. MPO activity was expressed as the delta absorbance units per minute (ΔA/min) in 5 μL of supernatant.

### Statistical analysis

To satisfy the assumptions of normality and the homogeneity of variances, the original values of S100A12 and MPO were transformed to a logarithmic scale. The normality of log-transformed values was then confirmed by Shapiro-Wilk test. The differences between S100A12 concentrations and MPO activities in different intestinal segments (duodenum, ileum, colon, and cecum) between dogs with CE and healthy Beagles were determined using Student’s *t*-tests. An analysis of variance (ANOVA) test was used to determine the association between S100A12 concentrations and MPO activities with total histopathological injury severity (insignificant, mild, moderate, or severe) and individual histopathological changes severity (normal, mild, moderate, or severe). A Spearman’s correlation coefficient was used to determine a potential correlation between S100A12 concentrations and MPO activities in dogs with CE and healthy Beagles. A possible correlation between CIBDAI score and S100A12 concentrations or MPO activities was tested using linear regression. The generalized logit-model was used to determine a possible association between S100A12 concentrations and MPO activities with clinical outcome (ie, FRE, ARE, SRE, or SNRE) in dogs with CE. All statistical differences were calculated based on the log-transformed values of S100A12 and MPO, and presented as mean and standard deviation in figures. However, untransformed values are presented as median (interquartile range: IQR) or median (range) as appropriate, for ease of interpretation in tables and texts. No adjustment was made for multiple comparisons in this study. For all analyses, we considered values of *p* < 0.05 as significant. All statistical analyses were performed using SAS 9.3 statistical software (SAS Institute Inc., Cary, NC, USA).

## Results

### Study population

Dogs with CE and healthy Beagles had a median age of 5 years (range 1-13 years) and 10.5 years (range 6-13 years), respectively. In the CE group, five dogs were intact females, nine dogs were spayed females, 15 dogs were intact males, and 11 dogs were castrated males. In the healthy Beagle control group, 10 dogs were intact females and eight dogs were intact males. The breeds of dogs with CE were the following: six mixed breed dogs, two German Shepherd dogs, two Parson Russell Terriers, two Shetland Sheepdogs, two Standard Poodles, two Rottweilers, and two Rough Collies. In addition, there were one of each of the following: Alaskan Malamute, Bichon Frise, Border Terrier, Chow Chow, Dalmatian, English Bulldog, Golden Retriever, Havanese, Irish Terrier, Jack Russell Terrier, Long-haired Dachshund, Mudi, Norwegian Lundehund, Rhodesian Ridgeback, Siberian Husky, Silky Terrier, Smooth Collie, Spanish Water Dog, Staffordshire Bull Terrier, Toy Poodle, West Highland White Terrier, and White Shepherd dog.

Out of 52 dogs, 12 dogs with chronic GI signs were excluded from further analysis. Seven dogs had gastrointestinal neoplasia (ie, 3 gastric adenocarcinomas, 2 lymphomas, 1 rectal plasma cell tumor, and 1 rectal adenocarcinoma). Four dogs had primary esophageal disorders, and one dog was positive for *Giardia* on fecal examination. A total of 40 dogs with CE were included in the study for analysis. Twenty-five dogs underwent gastroduodenoscopy, 10 dogs had both gastroduodenoscopy and colonoscopy, and colonoscopy alone was performed in 5 dogs. Sixty-eight intestinal mucosal biopsies were collected by endoscopy from four different segments of the intestinal tract of above-mentioned dogs, namely duodenum (*n* = 35), ileum (*n* = 12), colon (*n* = 15), and cecum (*n* = 6). Group distribution and inclusion/exclusion criteria of all dogs enrolled in this study are shown in Fig. 1.

### Mucosal S100A12 concentrations

**Fig. 2:**
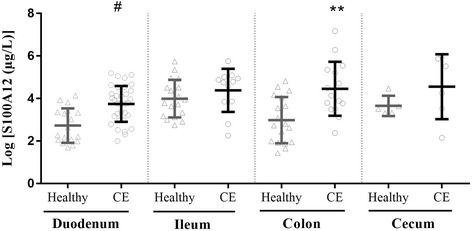
Scatter plot displaying log-transformed intestinal mucosal S100A12 concentrations in CE dogs and healthy Beagles. Data are expressed as the mean ± standard deviation. Individual data points are shown by triangles (samples from healthy Beagles) and circles (samples from CE dogs). CE: chronic enteropathies; **p < 0.01 vs. healthy colon; ^#^*p* < 0.0001 vs. healthy duodenum

Mucosal S100A12 concentrations in each intestinal segment in dogs with CE and healthy Beagles are shown in Fig. 2. The mucosal S100A12 concentrations in dogs with CE were significantly higher than those in healthy Beagles in the duodenum (43.93 [23.62–78.03] vs. 11.86 [7.66–29.1] μg/L; *p* < 0.0001) and colon (63.04 [33.53–211.53] vs. 15.94 [6.95–59.3] μg/L; *p* = 0.0011). Even though dogs with CE had higher mucosal S100A12 concentrations than healthy Beagles in the ileum (118.54 [47.26-142.9] vs. 48.1 [24.87-91.4] μg/L; p = 0.2725) and cecum (160.38 [43.27-326.28] vs. 33.53 [27.97-38.01] μg/L; p = 0.2194), these differences did not reach statistical significance. The lower detection limit of the ELISA assay for mucosal S100A12 was 0.2 μg/L.

### Mucosal myeloperoxidase activity

**Fig. 3:**
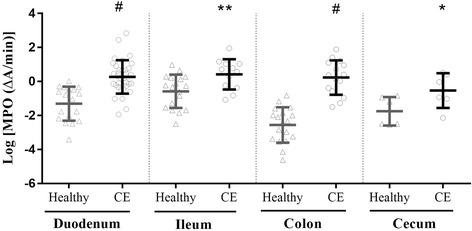
Scatter plot displaying log-transformed intestinal mucosal MPO activity in CE dogs and healthy Beagles. Data are expressed as the mean ± standard deviation. Individual data points are shown by triangles (samples from healthy Beagles) and circles (samples from CE dogs) CE: chronic enteropathies; *p < 0.05 vs. healthy cecum; **p < 0.01 vs. healthy ileum; ^#^*p* < 0.0001 vs. healthy duodenum and colon

Mucosal MPO enzyme activity in each intestinal segment in dogs with CE and healthy Beagles are shown in Fig. 3. The mucosal MPO activity of dogs with CE was significantly higher than the corresponding activity in healthy Beagles in the duodenum (1.3 [0.77–2.16] vs. 0.41 [0.11–0.64] ΔA/min; *p* < 0.0001), ileum (1.91 [0.72–2.83] vs. 0.75 [0.21–1.27] ΔA/min; *p* = 0.0083), colon (1.46 [0.57–3.01 vs. 0.09 [0.03–0.17] ΔA/min; *p* < 0.0001] ΔA/min), and cecum (0.68 [0.3–1.55] vs. 0.19 [0.08–0.4] ΔA/min; *p* = 0.0474). The lower detection limit of the spectrophotometric assay for mucosal MPO was 0.004 ΔA/min.

### Mucosal S100A12 concentrations and MPO activities in relation to histopathologic changes

Mucosal S100A12 concentrations showed a significant association with the severity of macrophage infiltration in the duodenum of dogs with CE (Table 1, *p* = 0.0439). In addition, the duodenal mucosal S100A12 concentrations were significantly higher if the inflammatory infiltrate contained neutrophils 61.1 [19.51-179.60] vs. 42.72 [7.41-102.46] μg/L; *p* = 0.037). However, when S100A12 concentrations were compared among the severity groups (normal, mild, moderate, and severe) of neutrophilic infiltration in the duodenum, we did not find a significant association in dogs with CE (Table 1, *p* = 0.1542). However, mucosal S100A12 concentrations were numerically higher in those with mild, moderate, or severe neutrophilic infiltration than in those with no neutrophilic infiltration. In addition, no significant association was found between S100A12 concentrations and severity of neutrophilic infiltration in the ileum, colon, and cecum of dogs with CE. In the colon, mucosal S100A12 concentrations showed a significant association with the severity of epithelial injury and total histopathologic injury (Table 2, *p* < 0.05). Higher mucosal MPO activity showed a significant association with severity of total histopathologic injury, epithelial injury, and eosinophilic infiltration in the duodenum (Table 1, *p* < 0.05). In the duodenum of dogs with CE, mucosal MPO activity was numerically higher in samples with moderate and severe neutrophilic infiltration than in samples with mild or no neutrophilic infiltration (Table 1). However, these differences did not reach statistical significance (*p* = 0.7326).

**Table 1:** Mucosal S100A12 and MPO levels in relation to histopathologic changes in the duodenum and ileum

Histopathological findings	n	Severity groups	S100A12 (μg/L*)* Median (IQR)	*P* value^a^	MPO (ΔA/min) Median (IQR)	*P* value^a^
Duodenum	35					
Total histopathological severity	35	Insignificant (*n* = 16)	33.65 (7.41-155.9)	0.2240	0.9 (0.2-2.95)	**0.006** ^**b**^
		Mild (*n* = 15)	53.12 (10.07-164.85)		1.49 (0.14-4.55)	
		Moderate (*n* = 4)	52.22 (42-179.6)		7.54 (0.94-17.16)	
Morphological criteria	35					
Villus stunting	35	Normal (*n* = 20)	44.18 (7.41-155.9)	0.4090	1.23 (0.14-4.55)	0.1439
		Mild (*n* = 10)	45.02 (19.51-179.6)		1.21 (0.37-3.88)	
		Moderate (*n* = 3)	56.27 (42.72-164.85)		2.16 (1.63-11.62)	
		Severe (*n* = 2)	26.04 (10.07-42)		8.85 (0.54-17.16)	
Epithelial injury	35	Normal (*n* = 30)	48.68 (7.41-164.85)	0.9122	1.23 (0.14-11.62)	**0.0455** ^**b**^
		Mild (*n* = 3)	28.94 (19.51-179.6)		1.58 (0.94-2.9)	
		Moderate (*n* = 2)	34.38 (26.76-42)		9.82 (2.48-17.16)	
Crypt distension	35	Normal (*n* = 29)	42.96 (7.41-164.85)	0.4156	1.29 (0.14-17.16)	0.0694
		Mild (*n* = 5)	67.87 (19.75-179.6)		0.94 (0.63-1.63)	
		Moderate (*n* = 1)	42.72		11.62	
Lacteal dilation	35	Normal (*n* = 19)	28.94 (7.41-155.9)	0.0889	1.24 (0.2-17.16)	0.0708
		Mild (*n* = 10)	54.7 (12.49-144.29)		1.07 (0.14-2.48)	
		Moderate (*n* = 6)	53.07 (42.72-179.6)		2.81 (0.94-11.62)	
Mucosal fibrosis	35	Normal (*n* = 31)	42.96 (7.41-179.6)	0.9191	1.36 (0.14-17.16)	0.6993
		Mild (*n* = 4)	63.88 (10.07-93.02)		0.83 (0.54-3.88)	
Inflammatory criteria	35					
Intraepithelial lymphocytes	35	Normal (*n* = 23)	42.96 (7.41-164.85)	0.9381	1.24 (0.14-17.16)	0.7695
		Mild (*n* = 10)	36.44 (11.53-179.6)		1.12 (0.37-4.55)	
		Moderate (*n* = 2)	53.07 (44.42-61.71)		2.41 (1.36-3.46)	
Lamina propria LPC	35	Normal (*n* = 2)	99.19 (42.48-155.9)	0.1963	0.53 (0.2-0.87)	0.0975
		Mild (*n* = 15)	24.83 (7.41-164.85)		1.24 (0.36-2.95)	
		Moderate (*n* = 15)	50.94 (10.07-179.6)		1.36 (0.14-11.62)	
		Severe (*n* = 3)	61.71 (42-93.02)		3.46 (0.77-17.16)	
Lamina propria eosinophils	35	Normal (*n* = 22)	58.99 (10.07-179.6)	0.0516	0.92 (0.14-3.46)	**0.0004** ^**b**^
		Mild (*n* = 5)	19.75 (7.41-42.96)		0.91 (0.64-2.95)	
		Moderate (*n* = 8)	43.33 (26.76-78.03)		3.39 (1.22-17.16)	
Lamina propria neutrophils	35	Normal (*n* = 20) Mild (*n* = 7) Moderate (*n* = 6) Severe (*n* = 2)	42.72 (7.41-102.46) 61.1 (19.51-155.9) 47.92 (24.83-179.6) 113.28 (61.71-164.85)	0.1542	1.07 (0.36-17.16) 1.49 (0.2-3.88) 1.4 (0.14-11.62) 2.81 (2.16-3.46)	0.7326
Lamina propria macrophage	35	Normal (*n* = 32)	42.84 (7.41-164.85)	**0.0439** ^**b**^	1.39 (0.14-17.16)	0.1786
		Mild (*n* = 3)	155.9 (44.2-179.6)		0.94 (0.2-1.36)	
Ileum	12					
Total histopathological severity	12	Insignificant (*n* = 8)	106.93 (9.59-317.21)	0.9816	1.91 (0.34-2.83)	0.3260
		Mild (*n* = 4)	125.92 (16.36-151.79)		2.3 (0.79-7.01)	
Morphological criteria	12					
Villus stunting	12	Normal (*n* = 7)	120.84 (9.59-317.21)	0.7443	2.04 (0.34-2.83)	0.3447
		Mild (*n* = 5)	116.24 (16.36-151.79)		1.77 (0.69-7.01)	
Epithelial injury	12	Normal (*n* = 10)	118.54 (9.59-317.21)	0.4956	1.91 (0.34-3.23)	0.2387
		Moderate (*n* = 2)	84.07 (16.36-151.79)		4.18 (1.36-7.01)	
Crypt distension	12	Normal (*n* = 11)	116.24 (9.59-317.21)	0.6913	2.04 (0.34-7.01)	0.4692
		Moderate (*n* = 1)	120.84		0.79	
Lacteal dilation	12	Normal (*n* = 8)	106.93 (9.59-317.21)	0.9881	1.56 (0.34-2.83)	0.2034
		Mild (*n* = 4)	125.92 (16.36-162.43)		2.63 (0.79-7.01)	
Mucosal fibrosis	12	Normal (*n* = 12)	118.54 (9.59-317.21)	–	1.91 (0.34-7.01)	–
Inflammatory criteria	12					
Intraepithelial lymphocytes	12	Normal (*n* = 6)	109.23 (9.59-162.43)	0.6809	1.42 (0.34-2.81)	0.1787
		Mild (*n* = 6)	123.62 (16.36-317.21)		2.3 (0.69-7.01)	
Lamina propria LPC	12	Normal (*n* = 4)	118.78 (47.08-162.43)	0.5561	2.12 (0.44-2.81)	0.9999
		Mild (*n* = 4)	68.6 (9.59-317.21)		1.81 (0.34-7.01)	
		Moderate (*n* = 4)	123.62 (47.32-151.79)		1.56 (0.69-3.23)	
Lamina propria eosinophils	12	Normal (*n* = 9)	130.99 (9.59-317.21)	0.2514	1.77 (0.34-3.23)	0.2019
		Mild (*n* = 2)	72.35 (47.08-97.62)		1.63 (0.44-2.81)	
		Moderate (*n* = 1)	16.36		7.01	
Lamina propria neutrophils	12	Normal (*n* = 9)	116.24 (9.59-317.21)	0.7430	2.04 (0.34-3.23)	0.5989
		Mild (*n* = 3)	120.84 (16.36-151.79)		1.36 (0.79-7.01)	
Lamina propria macrophage	12	Normal (*n* = 12)	118.54 (9.59-317.21)	–	1.91 (0.34-7.01)	–

Association between mucosal S100A12 concentrations and MPO activities with severity of histopathological findings in the duodenum and ileum of dogs with CE. Statistical differences were calculated based on the log-transformed values of S100A12 and MPO. Untransformed values are reported as median (IQR)

*n* sample size, *IQR* interquartile range, *LPC* lymphocytes/plasma cells

^a^ANOVA test after logarithmic transformation of original values

^b^statistically significant association

**Table 2:** Mucosal S100A12 and MPO levels in relation to histopathologic changes in the colon and cecum

Histopathological findings	n	Severity groups	S100A12 (μg/L) Median (IQR)	*P* value^a^	MPO (ΔA/min) Median (IQR)	*P* value^a^
Colon	15					
Total histopathological severity	15	Insignificant (*n* = 7)	49.01 (10.8-184.68)	**0.0274** ^**b**^	1.04 (0.22-3.37)	0.1280
		Mild (*n* = 7)	111.4 (32.81-538.8)		1.49 (0.38-4.08)	
		Moderate (*n* = 1)	1296.65		6.61	
Morphological criteria	15					
Epithelial injury	15	Normal (*n* = 8)	44.18 (10.8-184.68)	**0.0083** ^**b**^	1.28 (0.22-4.08)	0.4232
		Mild (*n* = 3)	254.57 (89.16-536.8)		1.49 (0.57-2.59)	
		Moderate (*n* = 3)	111.4 (32.81-211.53)		0.68 (0.38-3.01)	
		Severe (*n* = 1)	1296.65		6.61	
Crypt hyperplasia	15	Normal (*n* = 11)	89.61 (10.8-536.8)	0.8412	1.1 (0.22-3.37)	0.3437
		Mild (*n* = 3)	33.53 (32.81-1296.65)		1.46 (0.68-6.61)	
		Moderate (*n* = 1)	44.9		4.08	
Crypt dilation and distorsion	15	Normal (*n* = 12)	76.01 (10.8-536.8)	0.5706	1.48 (0.22-4.08)	0.5858
		Mild (*n* = 3)	49.01 (32.81-1296.65)		1.1 (0.68-6.61)	
Mucosal fibrosis and atrophy	15	Normal (*n* = 8)	53.97 (10.8-1296.65)	0.4267	0.86 (0.22-6.61)	0.9124
		Mild (*n* = 5)	111.4 (43.45-254.57)		2.59 (0.38-3.37)	
		Moderate (*n* = 1)	536.8		1.49	
		Severe (*n* = 1)	33.53		1.46	
Inflammatory criteria	15					
Lamina propria LPC	15	Normal (*n* = 2)	312.98 (89.16-536.8)	0.0554	1.03 (0.57-1.49)	0.4088
		Mild (*n* = 9)	49.01 (22.89-211.53)		1.1 (0.22-3.37)	
		Moderate (*n* = 3)	44.9 (10.8-254.57)		2.59 (0.28-4.08)	
		Severe (*n* = 1)	1296.65		6.61	
Lamina propria eosinophils	15	Normal (*n* = 9)	63.04 (10.8-254.57)	0.1076	1.04 (0.22-3.37)	0.1516
		Mild (*n* = 3)	33.53 (22.89-89.16)		1.46 (0.57-2.04)	
		Moderate (*n* = 3)	536.8 (44.9-1296.65)		4.08 (1.49-6.61)	
Lamina propria neutrophils	15	Normal (*n* = 10)	46.96 (10.8-1296.65)	0.2448	1.07 (0.22-6.61)	0.9400
		Mild (*n* = 4)	161.47 (33.53-254.57)		2.02 (0.38-3.01)	
		Moderate (*n* = 1)	536.8		1.49	
Lamina propria macrophage	15	Normal (*n* = 15)	63.04 (10.8-1296.65)	–	1.46 (0.22-6.61)	–
Cecum	6					
Total histopathological severity	6	Insignificant (*n* = 6)	160.38 (8.62-405.48)	–	0.68 (0.12-1.66)	–
Morphological criteria	6					
Epithelial injury	6	Normal (*n* = 3)	34.02 (8.62-249.74)	0.2187	0.37 (0.12-1.66)	0.4642
		Mild (*n* = 3)	351.79 (71.02-405.48)		0.97 (0.4-1.51)	
Crypt hypeplasia	6	Normal (*n* = 5)	71.02 (8.62-351.79)	0.3225	0.97 (0.12-1.66)	0.7163
		Mild (*n* = 1)	405.48		0.4	
Crypt dilation and distorsion	6	Normal (*n* = 5)	249.74 (8.62-405.48)	0.5229	0.98 (0.37-1.66)	0.0715
		Mild (*n* = 1)	34.02		0.12	
Mucosal fibrosis and atrophy	6	Normal (*n* = 6)	160.38 (8.62-405.48)	–	0.68 (0.12-1.66)	–
Inflammatory criteria	6					
Lamina propria LPC	6	Normal (*n* = 2)	211.41 (71.02-351.79)	0.6222	1.24 (0.97-1.51)	0.2594
		Mild (*n* = 4)	141.88 (8.62-405.48)		0.38 (0.12-1.66)	
Lamina propria eosinophils	6	Normal (*n* = 3)	34.02 (8.62-249.74)	0.2181	0.37 (0.12-1.66)	0.4642
		Mild (*n* = 3)	351.79 (71.02-405.48)		0.97 (0.4-1.51)	
Lamina propria neutrophils	6	Normal (*n* = 5)	71.02 (8.62-405.48)	0.4075	0.68 (0.12-1.66)	0.3664
		Moderate (*n* = 1)	351.79		1.51	
Lamina propria macrophage	6	Normal (*n* = 6)	160.38 (8.62-405.48)	–	0.68 (0.12-1.66)	–

Association between mucosal S100A12 concentrations and MPO activities with severity of histopathological findings in the colon and cecum of dogs with CE. Statistical differences were calculated based on the log-transformed values of S100A12 and MPO. Untransformed values are reported as median (IQR)

*n* sample size, *IQR* interquartile range, *LPC* lymphocytes/plasma cells

^a^ANOVA test after logarithmic transformation of original values

^b^statistically significant association

Based on the histological examination of intestinal samples from healthy Beagles, the median total WSAVA score of all samples was 0 (range 0–4), classifying all findings as insignificant.

### Mucosal S100A12 concentrations and MPO activities in relation to CIBDAI and clinical outcome

Based on treatment response after diet change or antibiotic or corticosteroid therapy, the outcomes of 30 dogs with CE were classified as follows: FRE (*n =* 10), ARE (*n =* 4), SRE (*n =* 13), and SNRE (*n =* 3). The median CIBDAI scores before and after treatment and the type of clinical outcome are summarized in Table 3. In dogs with CE, we did not observe any significant association between mucosal S100A12 concentrations or MPO activities with CIBDAI scores before treatment or the clinical outcome in each intestinal segment (data not shown, *p* > 0.05).

**Table 3:** Clinical outcome related to CIBDAI score before / after treatment in CE dogs

Clinical outcome	Number of dogs	CIBDAI before treatment Median (range)	CIBDAI after treatment Median (range)
Food-responsive enteropathy (FRE)	10	4 (2-9)	0 (0-1)
Antibiotic-responsive enteropathy (ARE)	4	5.5 (1-6)	0 (0-0)
Steroid-responsive enteropathy (SRE)	13	4 (0-7)	0 (0-1)
Steroid non-responsive enteropathy (SNRE)	3	7 (4-9)	9 (4-10)

Steroid non-responsive dogs (SNRE, *n* = 3) were euthanized because of severe clinical signs and unfavorable response to treatments. The median (range) of CIBDAI score before treatment was higher in SNRE dogs (*n* = 3) than in SRE dogs (*n* = 13) (7 [4–9] vs. 4 [0-7]). One SNRE dog and two SRE dogs had hypoalbuminemia (< 20 g/L). The median (range) of total histopathological injury score of SNRE dogs was 7 (5-7) and SRE dogs was 6 (1-11). The median (range) of mucosal S100A12 concentrations and MPO activities in SNRE dogs compared to SRE dogs were as follows in the duodenum: 53.12 (19.51-93.02) μg/L vs. 42.97 (12.49-179.6) μg/L and 1.22 (0.77-1.58) ΔA/min vs. 1.21 (0.14-17.6) ΔA/min, respectively.

### Mucosal S100A12 concentrations and MPO activities in relation to hypoalbuminemia in dogs with CE

Out of 40 dogs, 36 (90%) had normoalbuminemia with a median (range) serum albumin concentration of 32.7 g/L (24.9-39 g/L). However, four dogs (10%) had hypoalbuminemia with a median (range) serum albumin concentration of 12.2 g/L (11-13 g/L). Duodenal mucosal biopsies were taken from the four hypoalbuminemic dogs and mucosal S100A12 concentrations and MPO activities were evaluated. The mucosal S100A12 concentrations and MPO activities in hypoalbuminemic dogs with CE compared to normoalbuminemic dogs were as follows: 128.94 [69.54-175.91] g/L vs. 42.72 [23.62-61.1] g/L and 1.55 [0.81-3.14] ΔA/min vs. 1.58 [0.81-2.9] ΔA/min, respectively. Since the number of hypoalbuminemic dogs was too low for meaningful statistical analysis, we reported the results descriptively.

### Correlation between S100A12 concentrations and MPO activities

A moderate positive correlation between mucosal S100A12 concentrations and MPO activities was seen in dogs with CE and healthy Beagles combined (*r* = 0.392; *p* < 0.0001); this correlation was also observed in healthy Beagles alone (*r* = 0.327; *p* = 0.011). However, there was no correlation between mucosal S100A12 concentrations and MPO activities in dogs with CE alone (*p* = 0.273) or in the different intestinal segments from dogs with CE (duodenum: *p* = 0.0949; ileum: *p* = 0.245; colon: *p* = 0.265; cecum: *p* = 0.325).

## Discussion

To our knowledge, this paper is the first to report mucosal S100A12 concentrations and MPO activities in the intestines of dogs with CE. We observed that the mucosal S100A12 concentrations are significantly higher in dogs with CE than in healthy Beagles in the duodenum and colon. In the ileum and cecum, mucosal S100A12 concentrations were numerically higher in dogs with CE than those in healthy Beagles. However, this difference did not reach statistical significance. For the categories ileum and cecum which have currently the lowest case numbers, there would be the need for 95 and 27 dogs per group, respectively, to achieve 80% power. The results of our investigation in the duodenum are consistent with those from Leach et al. in humans with IBD [18]. In that study, duodenal and cecal biopsies from children with IBD (including UC, CD, or unclassified IBD) were cultured and supernatants were collected for ELISA determination of S100A12 concentrations. Similar to our results, children with UC, CD, or unclassified IBD had significantly higher S100A12 concentrations in the culture supernatants of duodenal and cecal mucosal biopsies than those in non-IBD control children [18]. In another similar study in humans, Foell et al. also found higher S100A12 concentrations in culture supernatants of colonic and ileal biopsy samples in patients with active CD than in those patients with inactive CD or healthy controls [15]. In addition, higher S100A12 concentrations were found in culture supernatants of colonic samples in patients with active UC than in those patients with inactive UC or healthy controls. Similar to Foell et al. [15], we found significantly higher S100A12 concentrations in colonic samples from dogs with CE than those in healthy Beagles. However, in contrast to people with CD, despite higher mucosal S100A12 concentrations in ileal samples of dogs with CE compared with healthy Beagles, this difference did not reach statistical significance. Heilmann et al. [24] previously reported significant upregulation of fecal S100A12 concentration in dogs with CE compared with healthy Beagles, which is consistent with our results in the duodenum and colon.

We found a significant association between colonic mucosal S100A12 concentrations and the severity of epithelial injury and total histopathological injury in dogs with CE. When S100A12 binds to RAGE, an inflammatory response occurs due to the production of pro-inflammatory cytokines via activation of NF-κB, which ultimately leads to tissue damage [9–11]. In the current study, we found higher mucosal S100A12 concentrations if the inflammatory infiltrate had a neutrophil or macrophage component. This is consistent with the findings in human patients with IBD [14]. However, since only 3 dogs had mild macrophage infiltration, the results should not be over interpreted and rather be a sign that more research needs to be performed with macrophage specific staining. In dogs with CE, however, Heilmann et al. [24] did not observe a significant association between fecal S100A12 and the presence of neutrophils and macrophages in intestinal mucosal biopsies or with the site(s) of inflammatory lesions. It is challenging to correlate fecal S100A12 concentrations to the site(s) of inflammatory lesions and cellular infiltrates within the GI tract, as it is unclear from which part of the intestine the S100A12 protein originates. Fecal S100A12 can be considered as reflective for mucosal S100A12 concentration giving an indication for the need to localize the site of the inflammatory lesions within the GI tract and to identify the areas most affected by the disease process. In addition to standard histology, mucosal biomarkers could provide valuable information concerning localization, severity and possible pathogenesis of inflammatory processes.

In our study, mucosal MPO activity was significantly higher in all studied segments of the intestine of dogs with CE than the corresponding activity in healthy Beagles. Similar results were also observed in the colonic mucosa of humans with IBD [31–34] and in animal models of human IBD [35–37]. In addition to the bactericidal activity of MPO products in activated phagocytes, there is considerable evidence that inappropriate or excessive stimulation of oxidant formation by this enzyme can result in host tissue damage [45]. In the present study, mucosal MPO activity showed a significant association with severity of epithelial injury and total histopathological injury in the duodenum of dogs with CE, which is consistent with findings in humans with IBD [32, 33] and also in animal models of human IBD [37]. The most severe histopathological injuries observed in mucosal samples with higher MPO activity could be explained by MPO-induced oxidative tissue damage in the inflamed mucosa. In addition, mucosal MPO activity showed a significant association only with eosinophil infiltration but not with neutrophil infiltration in the duodenum. In future studies, immunohistochemistry can be used to localize S100A12 and MPO cellular origin more specifically in the intestinal mucosa of dogs with CE.

We did not find a significant association between mucosal S100A12 concentrations or MPO activities in each intestinal segment with CIBDAI score before treatment or CE type (ie, FRE, ARE, SRE, or SNRE). Similar to our findings, mucosal S100A12 concentrations were not associated with the pediatric Crohn’s disease activity index (PCDAI) in children with IBD [18]. However, these findings contrast with the study of Heilmann et al. [24], who found a significant correlation between fecal S100A12 and the canine chronic enteropathy clinical activity index (CCECAI). It should be taken into consideration that CIBDAI or CCECAI include a number of subjective elements and consequently do not always represent the actual inflammatory burden, which may lead to discordance between the results of our study with others. In contrast to our results, another study by Heilmann et al. [26] revealed elevated fecal S100A12 concentrations in dogs with SRD compared with those with FRD or ARD. Elevated fecal S100A12 concentrations were also observed in SNRD dogs compared with those in dogs that underwent complete remission [26]. One of the limitations of our study is that we did not measure S100A12 concentrations in the feces of dogs with CE to assess for any associations between fecal S100A12 concentrations and types of clinical outcome. Accordingly, direct comparisons between our results and those with of Heilmann et al. [26] are not possible. One possible reason why a significant association between these biomarkers and clinical outcome was not observed might be the small number of samples from dogs with a certain clinical outcome (especially SNRE dogs, *n* = 3). Also the comparison between intestinal segments led to rather low case numbers when assessing the association between mucosal S100A12 concentrations and MPO activities with the type of clinical outcome. Another limitation is that the treatment follow up was not standardized in our study which should be considered when planning future studies.

Duodenal S100A12 concentrations were higher in four hypoalbuminemic dogs with CE. However, due to the low number of hypoalbuminemic dogs in our study, the clinical significance of our results is not clear. Heilmann et al. did not find a significant association between fecal S100A12 concentrations and hypoalbuminemia (< 20 g/L) in dogs with CE despite numerically higher fecal S100A12 concentrations in dogs with hypoalbuminemia [24]. Our findings suggest that further studies are warranted in more severely affected patients and should include measurements of both fecal and mucosal S100A12 concentrations.

We observed a moderate positive correlation between mucosal S100A12 concentrations and MPO activities in dogs with CE and healthy Beagles combined and also in healthy Beagles alone. However, no correlation was found in dogs with CE alone and also when analyzing different intestinal segments of dogs with CE. In pediatric studies, a moderate positive correlation was found between mucosal S100A12 and calprotectin concentrations only in non-IBD controls but not in patients with IBD [18]. Given that both S100A12 and MPO are derived predominantly from neutrophils, a correlation between the two biomarkers in dogs with CE was expected. Possible reasons for not having this correlation could be that two different methods were used to measure S100A12 concentrations and MPO activities and different functional pathways of these biomarkers in intestinal inflammation.

In the present study, we used stored intestinal mucosal samples from both diseased and healthy Beagle dogs. In the case of healthy Beagle dogs, the samples were collected during post-mortem examinations after finishing other non-related studies to avoid the unnecessary use of laboratory animals for experimental proposes. This methodology complies with the principles of replacement, reduction and refinement of animal experiments [46]. As this approach requires long-term stability of the measured analytes, we checked the MPO activity in 14 randomly chosen mucosal supernatant samples before and after 3 years of storage at − 80 °C. The median (range) of MPO activity at the first measurement (0.35 ΔA/min (0.01-1.92)) and after 3 years of storage at − 80 °C (0.35 ΔA/min (0.004-2.19)) were not statistically different (*P* = 0.101), which shows long-term stability of MPO activity in mucosal supernatant samples. Concentrations of S100A12 in fecal extracts stored frozen at − 80 °C have also been shown to be stable for years (Heilmann RM, unpublished data) and it is reasonable to assume that the same holds true for mucosal specimens. In addition, in our study, the control dogs were intact and older Beagles, however, it is rather unlikely that breed, gender, age, or being castrated or intact has an influence on our results. Age had also no influence on colonic mucosal MPO activity in baboons [47].

## Conclusions

This study showed that both mucosal S100A12 concentrations and MPO activities are increased in the in the duodenum and colon of dogs with CE compared with healthy Beagles; mucosal MPO activity is also increased in the ileum and cecum. In dogs with CE, mucosal S100A12 concentrations correlated with the severity of epithelial injury and total histopathological injury in the colon. Mucosal MPO activity is associated with the severity of epithelial injury and total histopathological injury in the duodenum of dogs with CE. The results provide supporting evidence for mucosal S100A12 and MPO as potential diagnostic biomarkers in dogs with CE. Further prospective research is needed to assess the value of measuring mucosal S100A12 concentrations and MPO activities in clinical practice, the relationship between mucosal and fecal S100A12 and MPO, and other inflammatory markers in dogs with CE.

## Abbreviations

ANOVA: Analysis of variance
ARD: Antibiotic-responsive diarrhea
ARE: Antibiotic-responsive enteropathy
BSA: Bovine serum albumin
CCECAI: Canine chronic enteropathy clinical activity index
CD: Crohn disease
CE: Chronic enteropathy
CIBDAI: Canine inflammatory bowel disease activity index
cTLI: Canine trypsin-like immunoreactivity
CV: Coefficients of variation
EDTA: Ethylenediaminetetraacetic acid
ELISA: Enzyme-linked immunosorbent assay
FRD: Food-responsive diarrhea
FRE: Food-responsive enteropathy
GI: Gastrointestinal
H_2_O_2_: Hydrogen peroxide
HE: Hematoxylin and eosin
HOCl: Hypochlorous acid
HTAB: Hexadecyltrimethylammonium bromide
IBD: Inflammatory bowel disease
ie: id est.
IQR: Interquartile range
MPO: Myelopreoxidase
NF-κB: Nuclear factor κB
O/E: Observed to expected ratios
PBS: Phosphate buffered saline
PCDAI: Pediatric crohn’s disease activity index
RAGE: Receptor for advanced glycation end products
SNRD: Steroid non-responsive diarrhea
SNRE: Steroid non-responsive enteropathy
SR: Steroid-responsive enteropathy
SRD: Steroid-responsive diarrhea
SRE: Steroid-responsive enteropathy
TBS: Tris buffered saline
UC: Ulcerative colitis
WSAVA: World small animal veterinary association
ΔA: Delta absorbance

## References

[1] Allenspach K, Wieland B, Grone A, Gaschen F (2007). Chronic enteropathies in dogs: evaluation of risk factors for negative outcome. J Vet Intern Med.

[2] Simpson KW, Pitfalls JAE (2011). Progress in the diagnosis and management of canine inflammatory bowel disease. Vet Clin North Am Small Anim Pract.

[3] Dandrieux JR (2016). Inflammatory bowel disease versus chronic enteropathy in dogs: are they one and the same?. J Small Anim Pract.

[4] Schmitz S, Glanemann B, Garden OA, Brooks H, Chang YM, Werling D (2015). A prospective, randomized, blinded, placebo-controlled pilot study on the effect of enterococcus faecium on clinical activity and intestinal gene expression in canine food-responsive chronic enteropathy. J Vet Intern Med.

[5] Cassmann E, White R, Atherly T, Wang C, Sun Y, Khoda S (2016). Alterations of the Ileal and colonic mucosal microbiota in canine chronic enteropathies. PLoS One.

[6] Meijer B, Hoskin T, Ashcroft A, Burgess L, Keenan JI, Falvey J (2014). Total soluble and endogenous secretory receptor for advanced glycation endproducts (RAGE) in IBD. J Crohns Colitis.

[7] Vogl T, Propper C, Hartmann M, Strey A, Strupat K, van den Bos C (1999). S100A12 is expressed exclusively by granulocytes and acts independently from MRP8 and MRP14. J Biol Chem.

[8] Shiotsu Y, Mori Y, Nishimura M, Sakoda C, Tokoro T, Hatta T (2011). Plasma S100A12 level is associated with cardiovascular disease in hemodialysis patients. Clin J Am Soc Nephrol.

[9] Foell D, Wittkowski H, Vogl T, Roth J (2007). S100 proteins expressed in phagocytes: a novel group of damage-associated molecular pattern molecules. J Leukoc Biol.

[10] Hofmann MA, Drury S, Fu C, Qu W, Taguchi A, Lu Y (1999). RAGE mediates a novel proinflammatory axis: a central cell surface receptor for S100/calgranulin polypeptides. Cell.

[11] Pietzsch J, Hoppmann S (2009). Human S100A12: a novel key player in inflammation?. Amino Acids.

[12] Dabritz J, Langhorst J, Lugering A, Heidemann J, Mohr M, Wittkowski H (2013). Improving relapse prediction in inflammatory bowel disease by neutrophil-derived S100A12. Inflamm Bowel Dis.

[13] de Jong NS, Leach ST, Day AS (2006). Fecal S100A12: a novel noninvasive marker in children with Crohn's disease. Inflamm Bowel Dis.

[14] Foell D, Kucharzik T, Kraft M, Vogl T, Sorg C, Domschke W (2003). Neutrophil derived human S100A12 (EN-RAGE) is strongly expressed during chronic active inflammatory bowel disease. Gut.

[15] Foell D, Wittkowski H, Ren Z, Turton J, Pang G, Daebritz J (2008). Phagocyte-specific S100 proteins are released from affected mucosa and promote immune responses during inflammatory bowel disease. J Pathol.

[16] Judd TA, Day AS, Lemberg DA, Turner D, Leach ST (2011). Update of fecal markers of inflammation in inflammatory bowel disease. J Gastroenterol Hepatol.

[17] Kaiser T, Langhorst J, Wittkowski H, Becker K, Friedrich AW, Rueffer A (2007). Faecal S100A12 as a non-invasive marker distinguishing inflammatory bowel disease from irritable bowel syndrome. Gut.

[18] Leach ST, Yang Z, Messina I, Song C, Geczy CL, Cunningham AM (2007). Serum and mucosal S100 proteins, calprotectin (S100A8/S100A9) and S100A12, are elevated at diagnosis in children with inflammatory bowel disease. Scand J Gastroenterol.

[19] Sidler MA, Leach ST, Day AS (2008). Fecal S100A12 and fecal calprotectin as noninvasive markers for inflammatory bowel disease in children. Inflamm Bowel Dis.

[20] Foell D, Kane D, Bresnihan B, Vogl T, Nacken W, Sorg C (2003). Expression of the pro-inflammatory protein S100A12 (EN-RAGE) in rheumatoid and psoriatic arthritis. Rheumatology (Oxford).

[21] Foell D, Seeliger S, Vogl T, Koch HG, Maschek H, Harms E (2003). Expression of S100A12 (EN-RAGE) in cystic fibrosis. Thorax.

[22] Lorenz E, Muhlebach MS, Tessier PA, Alexis NE, Duncan Hite R, Seeds MC (2008). Different expression ratio of S100A8/A9 and S100A12 in acute and chronic lung diseases. Respir Med.

[23] Tyden H, Lood C, Gullstrand B, Jonsen A, Nived O, Sturfelt G (2013). Increased serum levels of S100A8/A9 and S100A12 are associated with cardiovascular disease in patients with inactive systemic lupus erythematosus. Rheumatology (Oxford).

[24] Heilmann RM, Grellet A, Allenspach K, Lecoindre P, Day MJ, Priestnall SL (2014). Association between fecal S100A12 concentration and histologic, endoscopic, and clinical disease severity in dogs with idiopathic inflammatory bowel disease. Vet Immunol Immunopathol.

[25] Heilmann RM, Otoni CC, Jergens AE, Grutzner N, Suchodolski JS, Steiner JM (2014). Systemic levels of the anti-inflammatory decoy receptor soluble RAGE (receptor for advanced glycation end products) are decreased in dogs with inflammatory bowel disease. Vet Immunol Immunopathol.

[26] Heilmann RM, Volkmann M, Otoni CC, Grutzner N, Kohn B, Jergens AE (2016). Fecal S100A12 concentration predicts a lack of response to treatment in dogs affected with chronic enteropathy. Vet J.

[27] Myeloperoxidase KSJ (2005). Friend and foe. J Leukoc Biol.

[28] Roncucci L, Mora E, Mariani F, Bursi S, Pezzi A, Rossi G (2008). Myeloperoxidase-positive cell infiltration in colorectal carcinogenesis as indicator of colorectal cancer risk. Cancer Epidemiol Biomark Prev.

[29] Preiser JC (2012). Oxidative stress. JPEN J Parente Enteral Nutri.

[30] Odobasic D, Kitching AR, Semple TJ, Holdsworth SR (2007). Endogenous myeloperoxidase promotes neutrophil-mediated renal injury, but attenuates T cell immunity inducing crescentic glomerulonephritis. J Am Soc Nephrol.

[31] Hegazy SK, El-Bedewy MM (2010). Effect of probiotics on pro-inflammatory cytokines and NF-kappaB activation in ulcerative colitis. World J Gastroenterol.

[32] Kayo S, Ikura Y, Suekane T, Shirai N, Sugama Y, Ohsawa M (2006). Close association between activated platelets and neutrophils in the active phase of ulcerative colitis in humans. Inflamm Bowel Dis.

[33] Kruidenier L, Kuiper I, Van Duijn W, Mieremet-Ooms MA, van Hogezand RA, Lamers CB (2003). Imbalanced secondary mucosal antioxidant response in inflammatory bowel disease. J Pathol.

[34] Hansberry DR, Shah K, Agarwal P, Agarwal N (2017). Fecal myeloperoxidase as a biomarker for inflammatory bowel disease. Cureus.

[35] Li R, Chen Y, Shi M, Xu X, Zhao Y, Wu X (2016). Gegen Qinlian decoction alleviates experimental colitis via suppressing TLR4/NF-kappaB signaling and enhancing antioxidant effect. Phytomedicine.

[36] Lv J, Zhang Y, Tian Z, Liu F, Shi Y, Liu Y (2017). Astragalus polysaccharides protect against dextran sulfate sodium-induced colitis by inhibiting NF-kappacapital VE, Cyrillic activation. Int J Biol Macromolec.

[37] Kim JJ, Shajib MS, Manocha MM, Khan WI. Investigating intestinal inflammation in DSS-induced model of IBD. J Vis Exp. 2012;(60):e3678.10.3791/3678. 10.3791/3678 PMC3369627 22331082

[38] Hanifeh M, Heilmann RM, Sankari S, Rajamaki MM, Makitalo L, Syrja P (2015). S100A12 concentrations and myeloperoxidase activity in the intestinal mucosa of healthy dogs. BMC Vet Res.

[39] Day MJ, Bilzer T, Mansell J, Wilcock B, Hall EJ, Jergens A (2008). Histopathological standards for the diagnosis of gastrointestinal inflammation in endoscopic biopsy samples from the dog and cat: a report from the world small animal veterinary association gastrointestinal standardization group. J Comp Pathol.

[40] Washabau RJ, Day MJ, Willard MD, Hall EJ, Jergens AE, Mansell J (2010). Endoscopic, biopsy, and histopathologic guidelines for the evaluation of gastrointestinal inflammation in companion animals. J Vet Intern Med.

[41] Kilkenny C, Browne WJ, Cuthill IC, Emerson M, Altman DG (2010). Improving bioscience research reporting: the ARRIVE guidelines for reporting animal research. PLoS Biol.

[42] Jergens AE, Schreiner CA, Frank DE, Niyo Y, Ahrens FE, Eckersall PD (2003). A scoring index for disease activity in canine inflammatory bowel disease. J Vet Intern Med.

[43] Kilpinen S, Spillmann T, Syrja P, Skrzypczak T, Louhelainen M, Westermarck E (2011). Effect of tylosin on dogs with suspected tylosin-responsive diarrhea: a placebo-controlled, randomized, double-blinded, prospective clinical trial. Acta Vet Scand.

[44] Heilmann RM, Cranford SM, Ambrus A, Grutzner N, Schellenberg S, Ruaux CG (2016). Validation of an enzyme-linked immunosorbent assay (ELISA) for the measurement of canine S100A12. Vet Clin Pathol.

[45] Davies MJ (2011). Myeloperoxidase-derived oxidation: mechanisms of biological damage and its prevention. J Clin Biochem Nutr.

[46] Replacement FP (2002). Reduction and refinement. ALTEX.

[47] Tran L, Greenwood-Van Meerveld B (2013). Age-associated remodeling of the intestinal epithelial barrier. The journals of gerontology series a, biological sciences and medical. sciences.

